# Retraction: Mesons from Laser-Induced Processes in Ultra-Dense Hydrogen H(0)

**DOI:** 10.1371/journal.pone.0212979

**Published:** 2019-02-22

**Authors:** The PLOS ONE Editors

After publication, concerns were raised about the scientific validity of the results reported in this article [1]. The article was reassessed by a member of *PLOS ONE*’s Editorial Board and by two external reviewers with subject expertise who raised the following issues:

- The reported results are in conflict with the baryon number conservation law which forbids the production of the reported mesons. Claims of the presence of anti-baryons are not sufficiently supported by the evidence presented and are inconsistent with prior studies. The theoretical support published by other research groups cited in the article does not discuss the state of matter in question [2].
- The study’s detection methods and the reported observations are insufficient to support the article’s claims and are not sufficient to rule out other explanations. For example, a consulted reviewer advised that the reported meson decay time constants are consistent with “amplified electronics placed in the vicinity of intense laser irradiation experiments.”

Based on the advice received, and in the absence of sufficiently strong evidence to support the claims made, the *PLOS ONE* Editors retract this article. We regret that these issues were not identified prior to the article’s publication.

LH did not agree with retraction.
